# Tumor Necrosis Factor Induces Tumor Promoting and Anti-Tumoral Effects on Pancreatic Cancer via TNFR1

**DOI:** 10.1371/journal.pone.0075737

**Published:** 2013-09-30

**Authors:** Martin Chopra, Isabell Lang, Steffen Salzmann, Christina Pachel, Sabrina Kraus, Carina A. Bäuerlein, Christian Brede, Ana-Laura Jordán Garrote, Katharina Mattenheimer, Miriam Ritz, Stefanie Schwinn, Carolin Graf, Viktoria Schäfer, Stefan Frantz, Hermann Einsele, Harald Wajant, Andreas Beilhack

**Affiliations:** 1 Department of Internal Medicine II, Würzburg University Clinics, Würzburg, Germany; 2 Center for Interdisciplinary Clinical Research, Würzburg University, Würzburg, Germany; 3 Division of Molecular Internal Medicine, Department of Internal Medicine II, Würzburg University Clinics, Würzburg, Germany; 4 Department of Internal Medicine I, Würzburg University Clinics, Würzburg, Germany; 5 Comprehensive Heart Failure Center, Würzburg University, Würzburg, Germany; University of Tübingen, Germany

## Abstract

Multiple activities are ascribed to the cytokine tumor necrosis factor (TNF) in health and disease. In particular, TNF was shown to affect carcinogenesis in multiple ways. This cytokine acts via the activation of two cell surface receptors, TNFR1, which is associated with inflammation, and TNFR2, which was shown to cause anti-inflammatory signaling. We assessed the effects of TNF and its two receptors on the progression of pancreatic cancer by *in vivo* bioluminescence imaging in a syngeneic orthotopic tumor mouse model with Panc02 cells. Mice deficient for TNFR1 were unable to spontaneously reject Panc02 tumors and furthermore displayed enhanced tumor progression. In contrast, a fraction of wild type (37.5%), TNF deficient (12.5%), and TNFR2 deficient mice (22.2%) were able to fully reject the tumor within two weeks. Pancreatic tumors in TNFR1 deficient mice displayed increased vascular density, enhanced infiltration of CD4^+^ T cells and CD4^+^ forkhead box P3 (FoxP3)^+^ regulatory T cells (T_reg_) but reduced numbers of CD8^+^ T cells. These alterations were further accompanied by transcriptional upregulation of IL4. Thus, TNF and TNFR1 are required in pancreatic ductal carcinoma to ensure optimal CD8^+^ T cell-mediated immunosurveillance and tumor rejection. Exogenous systemic administration of human TNF, however, which only interacts with murine TNFR1, accelerated tumor progression. This suggests that TNFR1 has basically the capability in the Panc02 model to trigger pro-and anti-tumoral effects but the spatiotemporal availability of TNF seems to determine finally the overall outcome.

## Introduction

Pancreatic ductal adenocarcinoma (PDA) is one of the most devastating malignancies with exceptionally poor 5-year survival rates and very limited therapeutic options [1]–[3]. Various signaling pathways are perturbed in pancreatic cancer and this not only affects the tumor cells directly but also applies to the stromal cells within and around the tumor [4]–[6]. Especially NF-κB signaling is commonly deregulated in PDA [7]–[9]. A major activator of NF-κB is the cytokine tumor necrosis factor (TNF), which is mainly produced by activated immune cells, especially macrophages and T cells, but can also be expressed by tumor cells [10], [11].

TNF is a trimeric transmembrane type II protein from which a soluble form is released by proteolytic processing. The two forms of TNF interact with two receptors, TNFR1 and TNFR2, but differ in their ability to activate these receptors. Membrane-bound TNF strongly activates both receptors whereas soluble TNF, despite binding to TNFR2, only activates TNFR1 properly [12]. While TNFR1 is a typical representative of the death domain-containing subgroup of the TNF receptor protein family, TNFR2 belongs to the TRAF-interacting subgroup. Even though having a death domain, TNFR1 in response to TNF primarily initiates pro-inflammatory signaling pathways leading to the activation of NF-κB transcription factors and various MAP kinases but typically not in cell death induction. It is evident from the analysis of TNFR1 and TNFR2 knockout mice that many immunoregulatory processes are controlled by the two TNF receptors in an antagonistic, additive or even synergistic way but there is also evidence for autonomous functions of each of the two receptors [11], [13]. In particular, TNFR2 was shown to play an important role in the homeostasis of immunosuppressive regulatory T cells (T_regs_) [14]–[16].

In pancreatic cancer TNF plays a complex yet until now poorly understood role [17]–[23]. Here, we addressed how TNF and its receptors impact the immune control of PDA in an orthotopic syngeneic mouse model. Loss of TNFR1 within the host abrogated tumor control and resulted in enhanced tumor growth. TNFR1 deficiency caused deregulation of T cell infiltration and activation. We propose a novel anti-tumorigenic role of host TNFR1 in PDA where TNF-TNFR-interactions regulate the homeostasis of both regulatory and cytotoxic T cells deciding whether PDA is controlled and eventually rejected or grows progressively.

## Materials and Methods

### Ethics Statement

All experiments were performed according to the German regulations for animal experimentation. The study was approved by the Regierung von Unterfranken as the responsible authority (Permit Number 55.2-2531.01-76/10). All surgery was performed under esketamine/xylazine anesthesia, and all efforts were made to minimize suffering.

### Animals

C57Bl/6 deficient for TNF (B6.129S-Tnf^tm1Gkl^/J, short B6.TNF KO), TNFR1 (C57BL/6-Tnfrsf1a^tm1Imx^/J, short B6.TNFR1 KO), TNFR2 (B6.129S7-Tnfrsf1b^tm1Imx^/J, short B6.TNFR2 KO), TNFR1R2 (B6.129S-Tnfrsf1a^tm1Imx^Tnfrsf1b^tm1Imx^/J, short B6.TNFR1R2 KO) were initially obtained from Jackson Laboratories (Bar Harbor, ME, USA) and backcrossed to the albino C57Bl/6 background (C57BL/6J-Tyr<c-2J mice, Jackson Laboratories) for improved *in vivo* bioluminescence imaging sensitivity (reduced light absorption due to lack of melanin in the skin) as described previously [16]. Genotypes of KO mice were routinely checked by PCR. Female mice were used for experiments between 8 and 12 weeks of age. All mice were bred within the specified pathogen-free animal facility of the Center for Experimental Molecular Medicine of the University Hospital, Würzburg receiving rodent chow and autoclaved drinking water ad libitum.

### Cell Culture

For lentiviral transduction of Panc02 cells [24], 293 T cells were transiently transfected with a standard calcium phosphate precipitation protocol in 10 cm dishes with 10 µg pMDL and 5 µg RSV-REV packaging plasmids, 6 µg VSV/G envelope plasmid and 20 µg of the eGFP and firefly luciferase encoding plasmid FUGLW. Two days later, the supernatant containing the lentiviral particles was aspirated, filtered through a 0.45-µm filter, 8 µg polybrene/ml were added and the mixture was used to transduce the tumor cells. The transduced cells were flow sorted twice for eGFP-expression, termed hereafter Panc02-FUGLW. Cells were maintained in Dulbecco’s Modified Eagle’s Medium (DMEM) supplemented with 10% fetal bovine serum (FBS), 1% antibiotics (penicillin, streptomycin), L-glutamine and 0.1% β-mercaptoethanol. Cells were trypsinized and passaged twice weekly. Cell culture medium and supplements were obtained from Invitrogen (Darmstadt, Germany), all plastic ware was from Greiner BioOne (Frickenhausen, Germany). Panc02-FUGLW cells are syngeneic to C57BL/6 mice.

### Orthotopic PDA Model and In Vivo Bioluminescence Imaging

Panc02-FUGLW cells were trypsinized, harvested and washed twice with PBS. Recipient mice were anesthetized with i.p. injection of 80 mg/kg body weight (bw) esketamine hydrochloride (Pfizer, Berlin, Germany) and 16 mg/kg bw xylazine (cp Pharma, Burgdorf, Germany) and placed on a 37°C heating plate. Panc02-FUGLW cells were injected orthotopically as described elsewhere [17], [25] with slight modifications. Briefly, the abdominal cavity was opened by a minimal invasive transverse laparotomy. The head of the pancreas was identified and externalized. 1×10^4^ viable Panc02-FUGLW cells were slowly injected in 30 µl PBS into the head of the pancreas using a 710 RN 100 µl Hamilton syringe with a Gauge 28, 10 mm, Point Style 4 needle (Hamilton Syringe, Bonaduz, Switzerland). The pancreas was placed back into the abdominal cavity and the peritoneum and the skin were closed by running single-layer of 6-0 polyglactin sutures (Johnson & Johnson, Norderstedt, Germany).

For TNF treatment, mice were injected i.p. with 5 µg human TNF in 200 µl PBS every other day starting on the day of tumor cell inoculation. For *in vivo* bioluminescence imaging [26], mice were anesthetized and co-injected with 300 mg/kg bw D-luciferin (Biosynth, Staad, Switzerland). Ten minutes later, bioluminescence signals of the anesthetized mice were assessed with an IVIS Spectrum imaging system (Caliper Life Sciences, Mainz, Germany). Pictures were taken from the lateral view in automatic mode with a maximum exposure time of five minutes per picture. Pictures were evaluated using Living Image 4.0 software (Caliper Life Sciences).

### *Ex vivo* Imaging

On day 23 or 30 after tumor cell inoculation, mice were injected with D-luciferin and 10 minutes later euthanized. Internal organs were removed and subjected to *ex vivo* bioluminescence imaging [26]. Tissue samples were embedded in Tissue Tek OCT (Sakura Finetek, Staufen, Germany) for further histological analysis.

### Isolation of Immune Cells from Pancreatic Tissue and Spleens

Tissues were minced with a surgical blade and digested for 45 minutes at 37°C with 2 mg/ml collagenase D and 0.1 mg/ml DNase I (both from Roche, Mannheim, Germany). Tissue pieces were mashed through a 70 µm cell strainer and spun down. The cell pellet was resuspended in erythrocyte lysis buffer (168 mM NH_4_Cl, 10 mM KHCO_3_, 0.1 mM ethylenediaminetetraacetic acid (EDTA)) and incubated for 2 minutes. Then 10 volumes of PBS were added and the cells were spun down again. The resulting pellet was resuspended in PBS and cells were used for flow cytometry. Spleens were directly filtered through a 70 µm cell strainer into erythrocyte lysis buffer and washed once with PBS.

### Immunofluorescence Microscopy

Cryo-embedded tissues were cut into 3 µm thick sections on a Leica CM1900 cryostat (Leica Microsystems, Wetzlar, Germany) and mounted onto frosted slides. Slides were air-dried and fixed with acetone at room temperature for 7 minutes. Slides were washed and blocked with 2% FBS in PBS for 15 minutes. When biotin-conjugated antibodies were used, additional blocking using an Avidin/Biotin Blocking kit (Vector Laboratories, Burlingame, CA, USA) was performed. Slides were then incubated with the appropriate antibodies for 1 hour at room temperature. Between antibody-incubations, the slides were washed with PBS thrice. Slides were counterstained with DAPI and mounted with mounting medium (Vector Laboratories). Antibodies used were: CD11b-Alexa 647 (M1/70), CD31-Biotin (MEC13.3), CD4-Alexa 647 (GK1.5), CD8-Biotin (53-6.7), Foxp3-purified (FJK-16s) (eBioscience), donkey-anti-rat-Cy3 (Dianova, Hamburg, Germany), F4/80-Alexa 488 (CI:A3-1), GR-1-Biotin (RB6-8C5), streptavidin-Alexa 546 (Invitrogen). Images were obtained with a Zeiss Imager.Z1m fluorescence microscope (Carl Zeiss, Göttingen, Germany) and evaluated using Zeiss AxioVision software (Carl Zeiss). Immune cells were counted and given as cells/mm^2^ or as pixels/150 000 µm^2^ as assessed by Image J software (NIH, Bethesda, MD).

### Flow Cytometry

Cells were blocked with normal rat serum (1∶20 in PBS) and stained with appropriate antibodies at 4°C for 30 min. Following surface antigen staining, cells were washed once with PBS and labelled with propidium iodide. For intracellular staining, cells were stained with LIVE/DEAD fixable violet dead cell stain kit (Invitrogen) and further processed using the Mouse regulatory T cell staining kit #2 (eBioscience, Frankfurt, Germany) according to the manufacturer’s protocol. Antibodies used were from Biolegend (Uithoorn, The Netherlands) if not stated otherwise: α4β7-PE (DATK32), CCR4-APC (2G12), CCR5-PE (C34-3448), CCR7-APC (4B12), CD102-Biotin (3C4 (MIC2/4)), CD103-Pacific Blue (2E7), CD106-Alexa 488 (429), CD107a-FITC (1D4B), CD107b-Alexa 647 (M3184), CD11a-PE (M17/4) (BD), CD11b-Alexa 647 (M1/70), CD11b-PE/Cy7 (M1/70), CD11c-Alexa 647 (N418), CD25-PE (PC61.5) (eBioscience), CD24-PE (LG.7F9) (eBioscience), CD29-PE (HMβ1-1), CD4-FITC (RM4-5) (eBioscience), CD4-APC (RM4-5), CD44-PE (IM7), CD45.1-PE (A20), CD45.2-APC (104), CD49d-Alexa 647 (RI-2), CD49f-Alexa 488 (GoH3), CD54-PE (YN1/1.7.4), CD62E-PE (10E9.6) (BD), CD62L-APC/Cy7 (MEL-14), CD69-Pacific Blue (H1.2F3), CD8-PE/Cy7 (53-6.7), CXCR3-APC (CXCR3-173), CXCR4-Alexa 488 (2B11) (eBioscience), E-Selectin IgG fusion protein (R&D Systems, Wiesbaden, Germany), F4/80-Alexa 488 (C1:A3-1), Foxp3-APC (FJK-16s) (eBioscience), Ly6G-PE (1A8) (BD), P-Selectin IgG fusion protein (BD), TNFR1-APC (55R-286), TNFR2-PE (TR75-89), goat-anti-human IgG-PE (Jackson), streptavidin-Alexa 546 (Invitrogen). All experiments were performed on a BD FACS Canto II (BD) and sample data recorded using BD FACSDiva software and analyzed using FlowJo software (Tree Star, Ashland, OR, USA).

### RNA Isolation, Reverse Transcription and qRT-PCR

RNA was isolated from tissue samples using Qiashredder and RNeasy mini kit spin columns (Qiagen, Hilden, Germany). 1 µg total RNA was reverse transcribed using the QuantiTect Reverse Transcription Kit (Qiagen). Expression for genes of interest (primer sequences can be found in table 1) was analysed on an iCycler thermocycler (Bio-Rad, Munich, Germany) using iTaq Universal SYBR Green Supermix (Bio-Rad) and β-actin as reference gene. Expression levels were calculated using the ΔΔC_T_ method.

**Table 1:** Primer sequences used for qRT-PCR.

Gene	forward primer	reverse primer
eGFP (FUGW vector)	CAA GGG CGA GGA GCT GTT CA	CGT AGG TCA GGG TGG TCA CG
PD-1 (PDCD1: NM_008798.2)	ACA TCC TTG ACA CAC GGC GCA	TCT GGT TTG GGC GAG GGG CT
PDL-1 (PDCD1lg1: NM_021893.3)	CGC AGG CGT TTA CTG CTG CAT	TCA CGG GTT GGT GGT CAC TGT
Arginase 1 (NM_007482)	CTG TGA ACA CGG CAG TGG CT	CCC TTG GGA GGA GAA GGC GT
CTLA-4 (NM_009843.3)	ACC GCC ATA CTT TGT GGG CA	GGC TCT GTT GGG GGC ATT TT
Galectin 9 (Lgals9: NM_010708.2)	GTG CAG TAC CAA CAC CGC GT	TCC GTG GGA ACT GGA CTG GC
VEGF (NM_001025250.3+ NM_009505.4+ NM_001025257.3)	GCT GTA CCT CCA CCA TGC CA	TTA CAG CAG CCT GCA CAG CG
VEGF (NM_001110266.1+ NM_001110267.1+ NM_001110268.1)	GCT GGG TCA CTA ACC ACT GT	GTC TGC ATT CAC ATC TGC TG
IDO (NM_008324)	TGT GGC TAG AAA TCT GCC TG	CGC AGT AGG GAA CAG CAA TA
iNOS (NM_010927.3)	GGC AGC CTG TGA GAC CTT TG	GCA TTG GAA GTG AAG CGT TTC
TIM-3 (Havcr2: NM_134250.2)	CGG AGA GAA ATG GTT CAG AGA	TTC ATC AGC CCA TGT GGA AAT
GM-CSF (NM_009969.4)	GAG CAG GGT CTA CGG GGC AA	TTC AGA GCT GGC CTG GGC TT
TNFR1 (NM_011609.4)	GCT GGA GAT GCA GAA CGG GC	ACG AGG GGG CGG GAT TTC TC
TNFR2 (NM_011610.3)	GGA ACC TGG GTA CGA GTG CCA	GCG GAT CTC CAC CTG GTC AGT
TNF (NM_013693.2)	CCA CGT CGT AGC AAA CCA CC	GGT GAG GAG CAC GTA GTC GG
IL-2 (NM_008366.3)	CTC TGC GGC ATG TTC TGG ATT	CAG AAA GTC CAC CAC AGT TGC T
IL-12A (NM_008351.2)	CGT CGT GAC CAT CAA CAG GG	GTG CCA CCT TTG GGG AGA TG
Interferon gamma (NM_008337.3)	TCA GCA ACA GCA AGG CGA AA	TCT CTT CCC CAC CCC GAA TC
IL-4 (NM_021283.2)	GGT CTC AAC CCC CAG CTA GT	CCC TTC TCC TGT GAC CTC GT
IL-6 (NM_031168.1)	CCA TCC AGT TGC CTT CTT GGG	GGT CTG TTG GGA GTG GTA TCC T
IL-10 (NM_010548.2)	GAC TTT AAG GGT TAC TTG GGT TGC	ACT CTT CAC CTG CTC CAC TGC
IL-15 (NM_008357.2)	ATC GCC ATA GCC AGC TCA TC	ACC TAC ACT GAC ACA GCC CAA
IL-17A (NM_010552.3)	GCG GCT GAC CCC TAA GAA AC	ACA CGA AGC AGT TTG GGA CC

### *In vitro* TNF Treatment and Assessment of Metastatic Capabilities

1×10^5^ Panc02 cells per well were seeded in 6 well plates and left overnight. Cells were treated with 1.67 nM human TNF or 1.67 nM murine TNF for 48 h, harvested by gently scraping the monolayer off the plastic surface, washed twice with PBS and assessed for the expression of adhesion molecules and chemokine receptors by flow cytometry.

To test the capability of Panc02 cells to attach to different extracellular matrix components, the CytoSelect 48-well Cell Adhesion Assay (Cell Biolabs, San Diego, CA, USA) was used according to the manufacturer’s instruction. To test the capability of Panc02 cells to invade the basement membrane, the CytoSelect 24-Well Cell Invasion Assay (Cell Biolabs) was used according to the manufacturer’s instruction.

Untreated and TNF-treated Panc02 cells were further assessed for the activity of matrix metalloproteinases MMP-2 and MMP-9 by gelatin zymography. For protein isolation, myocardium tissue of an infarcted mouse heart as well as Panc02-FUGLW cell pellets were homogenized with Ripa buffer and PMSF (Cell Signaling, Frankfurt, Germany). The samples were separated on a 10% polyacrylamide gel containing 2.5 mg/ml gelatin at a constant voltage of 120 V for 2 h at 4°C. After electrophoresis, the proteins were renaturated by incubation of the gels in 2.5% Triton X-100 for 90 min at room temperature. The gels were then incubated in activation buffer (50 mM Tris-HCl, pH 7.5, 5 mM CaCl2, 0.2 M NaCl, and 0.02% Brij-35) for 12 h at 37°C. Finally, the gels were stained for 1 h with 0.5% coomassie blue staining solution and then destained in 40% v/v methanol, 10% v/v acetic acid to reveal bands of clearing which indicate proteolytic activity. The band intensity was quantified using ImageJ (version 1.44p).

### Statistics

All graphs shown are combined data from at least two independent experiments; the number of animals is indicated in the figure legends. All data are shown as mean ± standard error of mean. Figures were prepared using GraphPad Prism 5 software (La Jolla, CA, USA) and Adobe Photoshop 7 (San Jose, CA, USA). All groups were compared to the wild type or untreated control group, respectively by two-tailed unpaired student’s t-test using GraphPad InStat 3 software. Data reaching statistical significance are indicated as: * p ≤ 0.05, ** p ≤ 0.01.

## Results

### Loss of Host TNFR1 Abrogates Spontaneous Rejection of Orthotopic Panc02 Tumors

**Figure 1:**
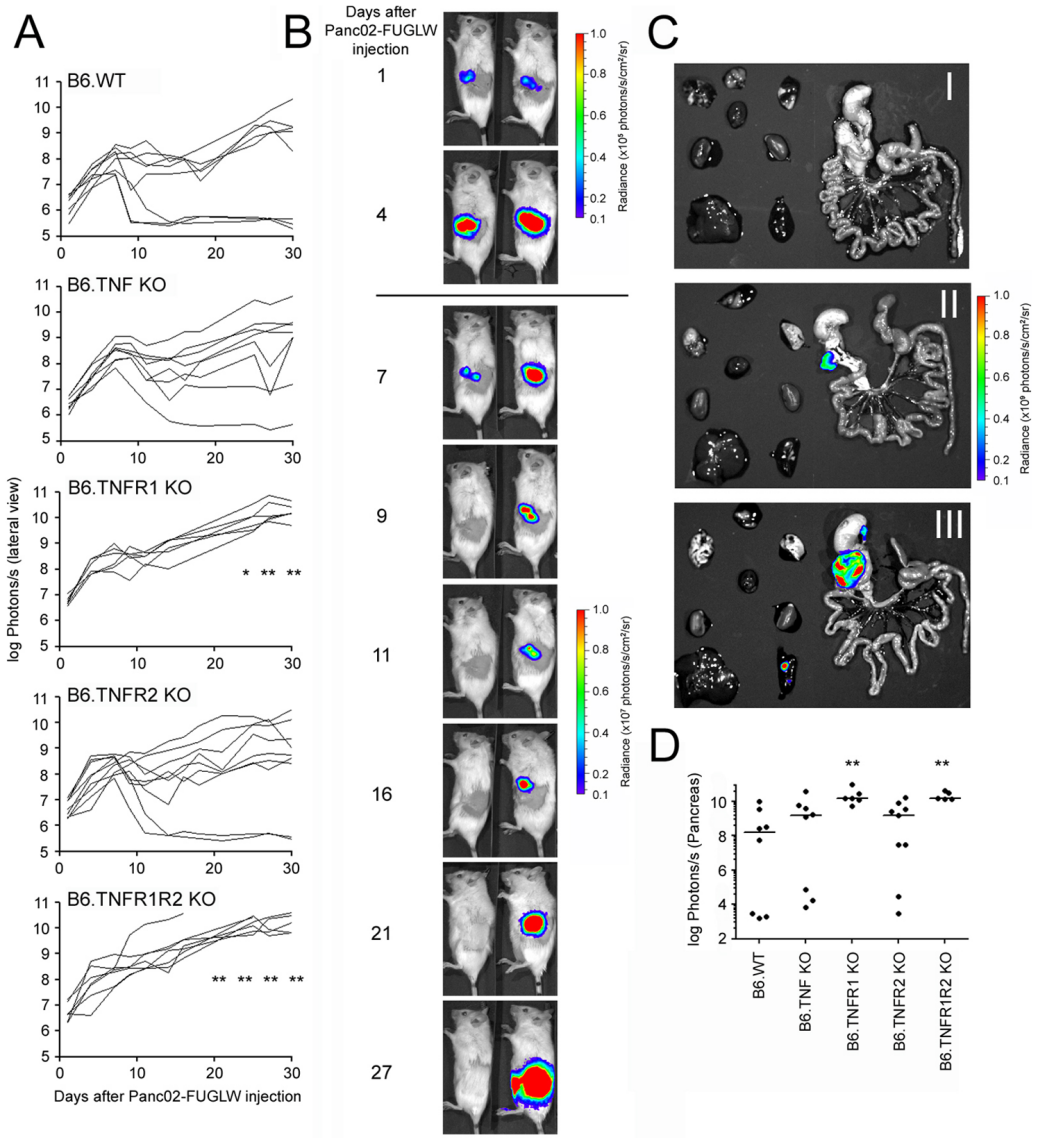
Loss of host TNFR1 abrogates spontaneous rejection of orthotopic Panc02 tumors. Murine pancreatic ductal adenocarcinoma (Panc02) cells were transduced to stably express eGFP and firefly luciferase and 10^4^ tumor cells were injected orthotopically into albino C57Bl/6 mice. Tumor growth in wild type mice (B6.WT) and mice that were deficient for TNF or its receptors was determined by *in vivo* BLI. A: Tumor growth displayed as total radiance (B6.WT n = 8, B6.TNF KO n = 8, B6.TNFR1 KO n = 6, B6.TNFR2 KO n = 9, B6.TNFR1R2 KO n = 7). B: Exemplary pictures of the imaging time course of a mouse that spontaneously rejected the tumor (left) and a mouse that could not control tumor progression (right). C: *Ex vivo* imaging one month after tumor cell inoculation. Internal organs were imaged for the presence of tumor cells. Exemplary pictures of a mouse that spontaneously rejected the tumor (I), a mouse with low tumor burden (II), and a mouse with high tumor burden (III). D: Pancreatic tumor size one month after Panc02 inoculation is displayed as total radiance (B6.WT n = 8, B6.TNF KO n = 8, B6.TNFR1 KO n = 6, B6.TNFR2 KO n = 9, B6.TNFR1R2 KO n = 5). * p≤0.05, ** p≤0.01. Combined data from four independent experiments.

In order to follow tumor growth non-invasively in a syngeneic mouse model of PDA, we generated Panc02 cells expressing eGFP and firefly luciferase (Panc02-FUGLW) and injected 1×10^4^ of these tumor cells orthotopically into albino wild type C57Bl/6 mice and albino C57Bl/6 mice deficient for TNF, TNFR1, TNFR2 or both TNFRs. In all genotypes assessed, Panc02-FUGLW tumors initially grew for the first 7 days following tumor inoculation (Figure 1A and B). 3/8 B6 wild type mice, 1/8 B6.TNF KO mice, and 2/9 B6.TNFR2 KO mice spontaneously rejected the tumor within 14 days. In contrast 13/13 mice deficient for TNFR1 or TNFR1 and TNFR2 could not reject the tumor. *In vivo* BLI (Figure 1A and B) and *ex vivo* BLI one month after tumor inoculation (Figure 1C and D) also revealed significantly higher tumor burden in mice deficient for TNFR1 (or TNFR1 and TNFR2) than in wild type mice. Thus, TNFR1 appeared as an important factor for the control and rejection of Panc02 tumors.

### Panc02 Tumors Show Altered T Cell Infiltration in B6.TNFR1 KO Mice

**Figure 2:**
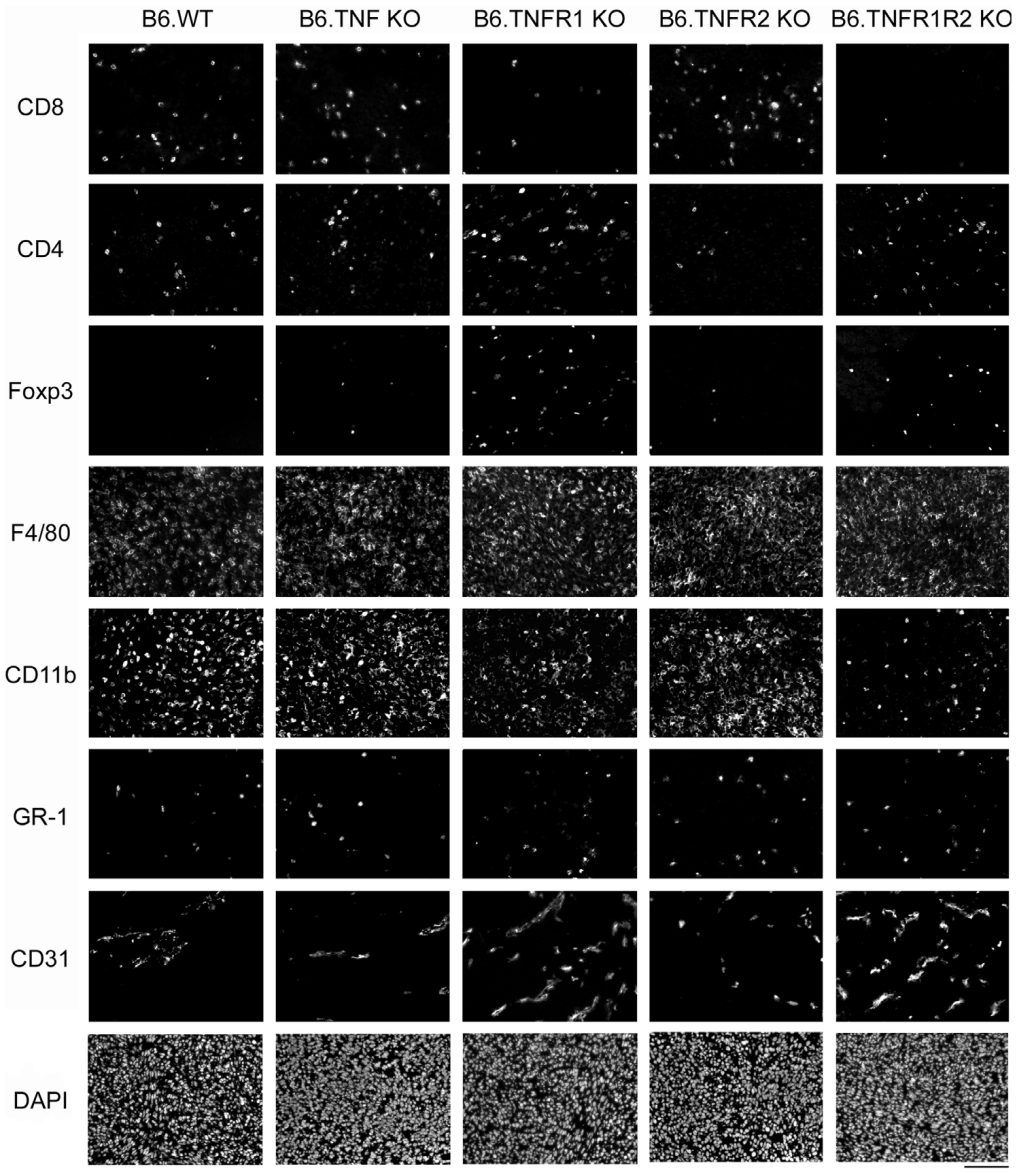
Loss of host TNFR1 perturbs the immunologic control of pancreatic ductal carcinoma. Panc02-tumors were explanted one month after tumor cell inoculation, consecutive histological sections were stained for indicated immune cell populations and blood vessels (CD31). Pancreatic tumors resulted in an influx of immune cell populations of the innate and adaptive immune system that were not observed in healthy pancreatic tissue under steady-state conditions. Of note, deficiency of TNFR1 resulted in a reduced cytotoxic CD8^+^ T cell infiltration but increased T_reg_ cell infiltration. Exemplary photomicrographs are shown. Scale bar indicates 100 µm.

**Figure 3:**
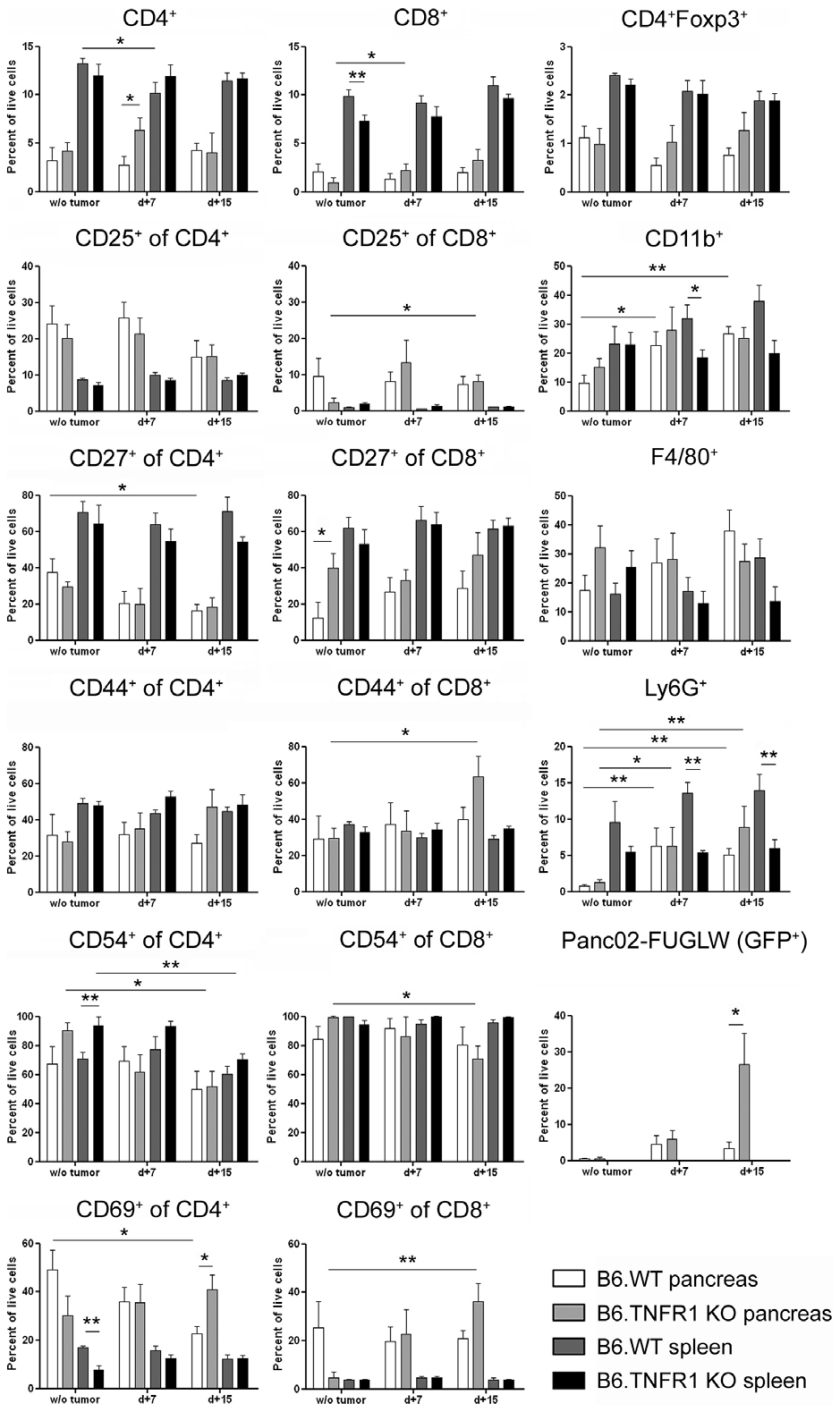
Loss of host TNFR1 does not affect the activation status of tumor-infiltrating T cells. Pancreata and spleens from naïve and tumor-bearing mice one and two weeks after tumor cell inoculation were explanted and prepared as single cell suspensions. T cells were analyzed for the expression of activation-associated surface receptors by flow cytometry. Furthermore, the percentage of T_regs_, myeloid cells and tumor cells was determined by flow cytometry (w/o tumor: B6.WT n = 8, B6.TNFR1 KO n = 6; d+7: B6.WT n = 7, B6.TNFR1 KO n = 6; d+15: B6.WT n = 7, B6.TNFR1 KO n = 7). *p≤0.05, **p≤0.01. Combined data from four independent experiments.

Next, we analyzed immune cell infiltration into Panc02-FUGLW tumors grown in either C57Bl/6 wild type or C57Bl/6 mice deficient for TNF, TNFR1 or TNFR2 (Figure 2 and Table 2) to address whether the loss of tumor control might be related to changes in the immune cell infiltrate of the tumor. Loss of TNFR1 correlated with decreased numbers of infiltrating CD8^+^ T cells and increased numbers of infiltrating CD4^+^Foxp3^+^ T_regs_. TNFR1 deficiency furthermore significantly increased vascular density as assessed by CD31-expression but did not significantly modify infiltration with innate immune cells of the myeloid lineage (CD11b^+^, F4/80^+^, Gr1^+^ cells). The altered T cell infiltration patterns of Panc02-FUGLW tumors from B6.TNFR1 KO one month after inoculation prompted us to assess the T cell phenotype in the early stage of tumor expansion. Therefore, we analyzed the expression of activation markers on CD4^+^ and CD8^+^ T cells 7 and 15 days after tumor cell injection (Figure 3). T cell activation markers only subtly differed. Yet, we observed a trend towards higher activation with time of CD8^+^ T cells (expression of CD25 and CD69) and lower activation of CD4^+^ T cells (expression CD27, CD54, and CD69). Tumor growth was strongly associated with myeloid inflammation. Flow cytometry revealed increased percentages of CD11b^+^ and Ly6G^+^ cells in the pancreas over time independent of the genetic background of tumor bearing mice. Interestingly, on a systemic level, loss of TNFR1 resulted in significantly decreased CD11b^+^ and Ly6G^+^ cell numbers in the spleens of tumor-bearing mice. Increased tumor growth within pancreata of TNFR1 KO mice was confirmed by significantly elevated numbers of eGFP^+^ Panc02-FUGLW cells in these mice two weeks after tumor inoculation (Figure 3).

**Table 2:** Loss of host TNFR1 perturbs the immunologic control of Panc02 tumors.

Genotype	CD8+(mm^−2^)	CD4^+^(mm^−2^)	T_regs_(mm^−2^)	F4/80^+^(densitiy×10^4^)	CD11b^+^(densitiy×10^4^)	CD11b^+^GR-1^+^(mm^−2^)	CD31^+^(densitiy×10^3^)
B6.WT	158.9±14.2	149.4±83.2	42.2±23.0	29.6±5.6	14.9±4.8	171.7±70.2	20.2±2.7
B6.TNF KO	209.8±36.5	208.4±79.1	72.4±29.1	29.6±2.1	8.8±2.1	186.7±97.3	15.5±2.9
B6.TNFR1 KO	127.1±26.7	374.7±62.6	177.8±35.4*	30.9±7.2	7.0±3.3	167.6±53.0	26.2±5.7
B6.TNFR2 KO	262.6±59.6	100.7±21.8	35.2±7.6	29.3±1.5	9.9±3.1	201.1±73.0	19.1±3.1
B6.TNFR1R2 KO	64.4±27.1*	295.6±155.7	116.1±52.8	29.6±6.7	4.6±1.9	122.2±35.3	42.9±3.9**

Panc02-tumors were explanted one month after tumor cell inoculation, sectioned, and stained for different immune cells and blood vessels (B6.WT n = 4, B6.TNF KO n = 5, B6.TNFR1 KO n = 5, B6.TNFR2 KO n = 6, B6.TNFR1R2 KO n = 4).

* p≤0.05, ** p≤0.01.

**Figure 4:**
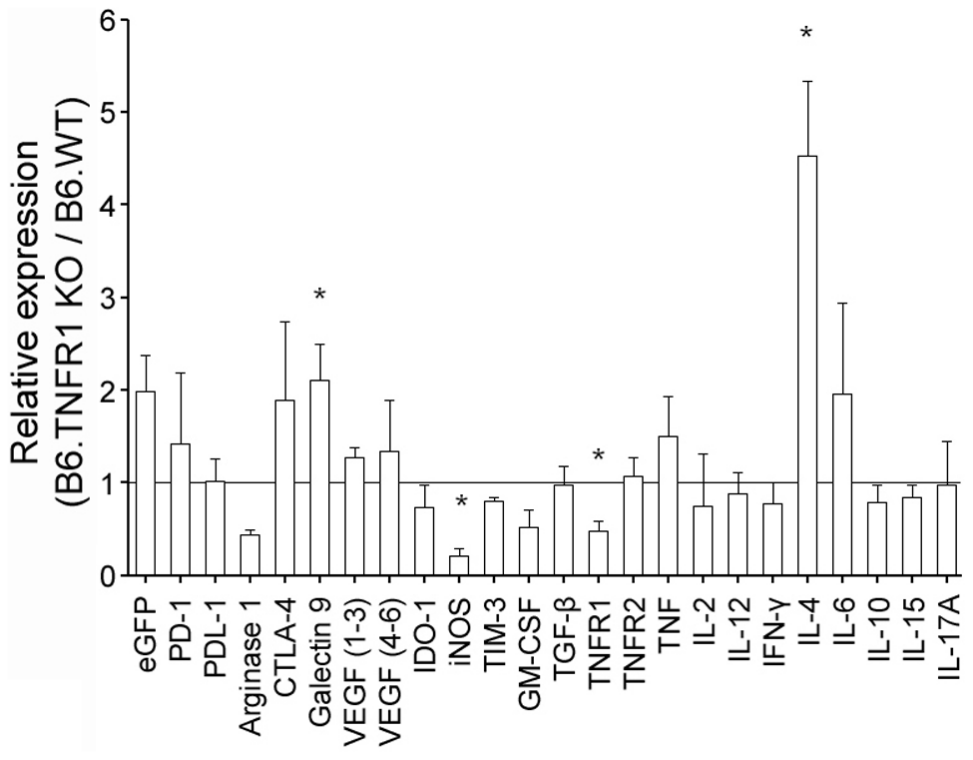
Loss of host TNFR1 affects the expression of immunosuppressive genes and IL-4. Panc02-tumors were explanted one month after tumor cell inoculation and total RNA was isolated from the tumor tissue. RNA was reverse transcribed and amplified by qRT-PCR. Data is presented as relative expression within tumors derived from B6.TNFR1 KO mice compared to tumors derived from wild type (WT) mice (B6.WT n = 3, B6.TNFR1 KO n = 4). * p≤0.05.

To further assess the mechanistic differences of tumor control between wild type and TNFR1 KO mice, we assessed the expression of immunosuppressive genes and various cytokines in Panc02-FUGLW tumors (Figure 4). Here, we observed on a transcriptional level significantly increased expression of Galectin-9 and IL-4 while the expression of iNOS was significantly reduced.

### Exogeneous TNF does not Induce Metastasis Despite Enhancing Tumor Growth and Influencing T_reg_ Homeostasis

**Figure 5:**
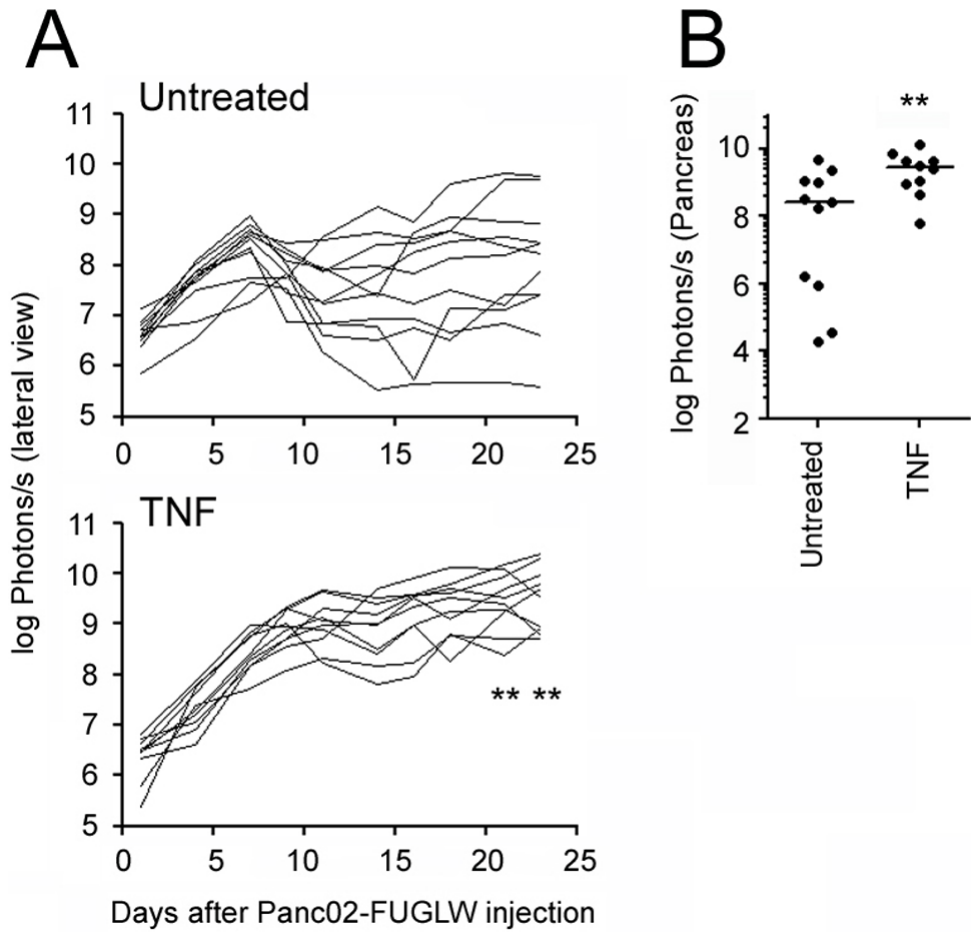
Exogenous TNF treatment increases orthotopic Panc02 tumor growth. 10^4^ tumor cells were injected into the spleen of albino B6.WT mice. The mice were either left untreated or were treated every other day with 5 µg of recombinant human TNF. Tumor growth was determined by *in vivo* BLI. A: Tumor growth displayed as total radiance (untreated n = 11, TNF n = 10). B: *Ex vivo* imaging 23 days after tumor cell inoculation. Internal organs were imaged for the presence of tumor cells. Pancreatic tumor size is displayed as total radiance (untreated n = 11, TNF n = 10). ** p≤0.01. Combined data from two independent experiments.

TNF was shown to enhance tumor cell metastasis in several *in vivo* mouse models [27]–[29] including an orthotopic xenotransplantation model of PDA with pancreatectomy-induced metastasis [17]. To test whether TNF also influences Panc02-FUGLW tumor growth and metastasis, we treated tumor bearing mice with recombinant human TNF, which only binds to murine TNFR1, every other day for three weeks following tumor cell inoculation. This significantly increased overall tumor growth within pancreata and ablated spontaneous tumor rejection as assessed with *in vivo* and *ex vivo* BLI (Figure 5A and B) but did not induce metastasis to the liver, the kidneys, or the mesentery (data not shown). We also analyzed the infiltration of CD8^+^ and CD4^+^ T cells, and T_regs_ into tumors of untreated and TNF-treated animals and indeed observed that human TNF treatment significantly increase T_reg_ numbers within the tumors (Table 3). Since we and others have found previously that TNFR2 rather than TNFR1 on T_regs_ is required to drive their expansion [14]–[16], this points to an indirect mechanism by which administration of human TNF triggers T_reg_ expansion in our PDA model.

**Table 3:** Exogenous TNF treatment perturbs the immunologic control of Panc02 tumors.

Treatment	CD8^+^ (mm^−2^)	CD4^+^ (mm^−2^)	T_regs_ (mm^−2^)	CD31^+^ (densitiy×10^3^)
Untreated	342.9±80.6	302.9±65.1	86.0±23.8	12.3±1.8
TNF	376.3±88.9	425.2±70.9	158.8±22.9 *	14.3±2.1

Panc02-tumors from untreated and human TNF treated mice were explanted 23 days after tumor cell inoculation, sectioned, and stained for different immune cells and blood vessels (untreated n = 7, TNF n = 9). * p≤0.05.

### Panc02 Cells Show Little In Vitro Capabilities for Metastasis

**Figure 6:**
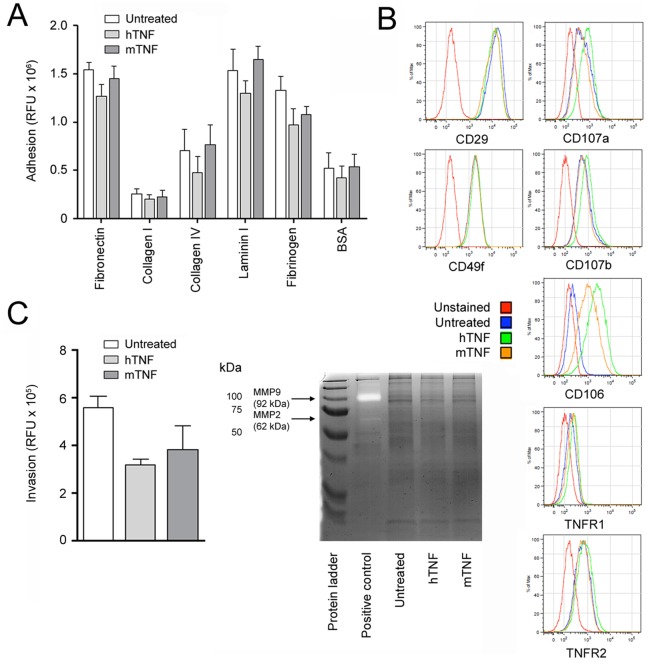
Panc02 cells show little *in vitro* capabilities for metastasis. Panc02 cells were treated with 1.67: Adhesion to different extracellular matrix proteins (n = 4). B: Flow cytometric determination of the expression of proteins involved in adhesion and migration (n = 3). C: Invasive capabilities of Panc02 cells. Left panel: *In vitro* invasion of the basement membrane (n = 3). Right panel: Gelatin zymography of tumor cell samples. Infarcted mouse heart lysate was used as a positive control (n = 4).

Due to the limited metastatic activity of Panc02 cells *in vivo*, we analyzed the metastatic capabilities of these cells *in vitro* particularly also upon TNF stimulation. Panc02 cells adhered to extracellular matrix proteins fibronectin, laminin I, and fibrinogen, but neither to collagen I nor IV. Neither human nor murine TNF modified adhesion of Panc02 cells to extracellular matrix components (Figure 6A) despite strong triggering of the classical NF-κB pathway (data not shown). Next, we analyzed Panc02 cells for their expression of proteins involved in adhesion and migration of cells (Figure 6B). We found the PDA tumor cells to express CD29 (integrin β1), CD49f (integrin α6), and CD107a and b, the expression of which was not modulated by TNF. CD106 (VCAM-1) expression, however, was induced by TNF. We also assessed the invasive capability of Panc02 cells by using an *in vitro* invasion assay and measuring gelatinolytic activities (Figure 6C). TNF did not significantly induce invasion and whereas infarcted mouse myocardium showed MMP-9 and -2 activities, Panc02 cells did not, irrespective of TNF stimulation. In sum, the results of the *in vitro* analysis of metastasis-related factors confirmed the limited metastatic activity observed with Pan02 cells *in vivo*.

## Discussion

Here we demonstrated that TNFR1 is an important receptor for the immunologic control of PDA. Loss of this receptor within the host results in perturbations of immune cell infiltration into the tumor and ablates immunosurveillance mechanisms. Nevertheless, exogenous activation of TNFR1 with recombinant TNF in tumor-bearing animals resulted in enhanced tumor progression, speaking for a double-edged role of TNF-TNFR1-interactions in PDA tumor control.

A recent study analyzed T antigen-induced multistage carcinogenesis in pancreatic islets and found that while in wild type mice infiltrating CD4^+^ T cells induced tumor dormancy, loss of TNFR1 on these cells switched them to a tumor-promoting phenotype by stimulating angiogenesis [18]. These observations are in line with our results, i.e. the loss of TNFR1 increased vascular densities within the tumors. While TNFR1-deficiency did not consistently modify the activation phenotype of tumor-infiltrating T cells, we found more CD4^+^ T cells infiltrating the tumors in TNFR1-deficient than in wild type mice. Furthermore, we observed decreased numbers of infiltrating CD8^+^ T cells and increased numbers of T_regs_. Chee and colleagues found the loss of TNFR1 in NOD mice to protect them from diabetes by affecting the homing of conventional T cells into pancreatic islets while increasing the numbers of T_reg_ cells [30]. In our model, TNFR1 deficiency resulted in changes in the expression of IL-4, Galectin-9, and iNOS. In line with this, IL-4 has been described as a potent T_H_2 cytokine [31] that was proposed to promote PDA tumor cell growth and thereby play an active role in tumor progression of this malignancy [32], [33]. Galectin-9 suppresses T_H_1 immune functions [34] and induces T_regs_ [35]. The role of this protein in PDA is not well established, nevertheless, increased expression of both IL-4 and Galectin-9 might hint towards a mechanism explaining increased CD4^+^ T cell and T_reg_ infiltration into Panc02 tumors grown in TNFR1-deficient mice. iNOS was described to be upregulated in pancreatic tumors [36], [37].

The role of TNF in pancreatic tumor progression is controversial. While some studies demonstrated anti-tumorigenic properties of TNF [19], [21], others have shown the opposite results [17], [20]. We demonstrate here a pro-tumorigenic function of systemically active TNF as the treatment of tumor-bearing mice with this cytokine significantly increased tumor growth. This goes along with elevated numbers of T_regs_ within the tumors. We have recently shown a similar mechanism in the B16F10 pulmonary metastasis model [16] where exogenous TNF treatment enhanced tumor growth in a T_reg_-dependent manner.

TNF is known to play a promoting role in cancer metastasis [16]–[17], [27]–[29]. Despite this, we did not observe increased metastasis of the primary Panc02 tumor to the liver upon exogenous TNF treatment. Whereas PDA readily metastasizes in other models [17], [38] and in human patients [39], [40], the Panc02 tumor does not appear to have the capabilities to metastasize spontaneously. The tumor cells showed very little gelatinolytic capacity and *in vitro* neither their invasive nor their adhesive capabilities were modulated by TNF stimulation.

In summary, we propose a role of TNFR1 in tumor immunosurveillance of PDA. While our data speak for an immune mediated anti-tumorigenic effect of TNF via TNFR1, as cautionary result we also observed that the exogenous treatment of tumor-bearing mice with TNF augmented tumor growth rather than controlled it.
